# Nanomaterials for Biomedical Applications: Production, Characterisations, Recent Trends and Difficulties

**DOI:** 10.3390/molecules26041077

**Published:** 2021-02-18

**Authors:** Mostafa Mabrouk, Diganta B. Das, Zeinab A. Salem, Hanan H. Beherei

**Affiliations:** 1 Refractories, Ceramics and Building Materials Department, National Research Centre, 33El Bohouth St (former EL Tahrir St), Dokki, Giza P.O. 12622, Egypt; hananh.beherei@gmail.com; 2 Department of Chemical Engineering, Loughborough University, Loughborough LE113TU, Leicestershire, UK; 3 Department of Oral Biology, Faculty of Dentistry, Cairo University, Giza P.O. 12613, Egypt; zeinab.salem@dentistry.cu.edu.eg; 4 Faculty of Oral and Dental Medicine, Ahram Canadian University, 6 October City P.O. 12573, Egypt

**Keywords:** tissue regeneration, drug delivery systems, preparation methods/tools, characterisation techniques

## Abstract

Designing of nanomaterials has now become a top-priority research goal with a view to developing specific applications in the biomedical fields. In fact, the recent trends in the literature show that there is a lack of in-depth reviews that specifically highlight the current knowledge based on the design and production of nanomaterials. Considerations of size, shape, surface charge and microstructures are important factors in this regard as they affect the performance of nanoparticles (NPs). These parameters are also found to be dependent on their synthesis methods. The characterisation techniques that have been used for the investigation of these nanomaterials are relatively different in their concepts, sample preparation methods and obtained results. Consequently, this review article aims to carry out an in-depth discussion on the recent trends on nanomaterials for biomedical engineering, with a particular emphasis on the choices of the nanomaterials, preparation methods/instruments and characterisations techniques used for designing of nanomaterials. Key applications of these nanomaterials, such as tissue regeneration, medication delivery and wound healing, are also discussed briefly. Covering this knowledge gap will result in a better understanding of the role of nanomaterial design and subsequent larger-scale applications in terms of both its potential and difficulties.

## 1. Introduction

Nano (n) is a prefix that is used to describe 10^−9^ of any parameter. For example, the nucleus diameter of lead (Pb) is ~0.35 nm. The idea behind nanotechnology was presented by the famous material scientist Richard, P. Feynman in his seminal lecture entitled There’s Plenty of Room at the Bottom in the December 1959 Meeting of the American Physical Society [1]. From that time onwards, there have been numerous advancements in material science that have exhibited Feynman’s vision of controlling material properties at the atomic scale. In 1974, Norio Taniguchi, an educator at the Tokyo University of Science (Japan), coined the term nanotechnology to depict high-accuracy and ultra-fine measurements. Taniguchi presented a top-down theory by foreseeing upgrades and scaling down in coordinated circuits, optoelectronic tools, mechanical devices and personal computer (PC) memory chips [2]. Around 10 years later, K. Eric Drexler presented a bottom-up design approach when he examined the formation of bigger particles from their atomic and sub-atomic scales as another possible direction of nanotechnology [3].

Nanotechnologies have been shown to be beneficial for different specialties that include drug delivery, purification of water, data management and the creation of nanoscale materials for healthcare and other industrial applications. The technologies involve the creation and control of material properties at the nanometre scale either by scaling up from a single collection of nanoparticles (NPs) or by refining/diminishing bulk materials into a desired nanoscale [4]. Nanotechnologies also deal with the arrangement, depiction, design and usages of structures, devices and systems by controlling the shape and size of particles in nanometre-scale materials such as NPs [5]. It is clear that nanotechnologies have now evolved for numerous critical uses, ranging from natural sciences to biomedicines and space innovations [6]. Today, an assortment of nanoscale-organised materials and instruments can be developed and applied. Nanotechnologies have also provoked the ascent of another field termed nanomedicine, which predominantly upgrades or changes the remedial discovery, treatment and prevention of diseases on the cellular level of the human body [7].

The greater part of these approaches incorporates the connection of nanostructured materials with biological subjects. The capacity of methodically altering the properties of these nanomaterials by controlling their structures and their properties at the nanoscale makes them a highly alluring contender for use in the diagnosis, treatment and regeneration of malfunctional biological systems [8]. Additionally, different nanostructures have been explored to evaluate their properties and conceivable applications in biosensors. These structures incorporate nanotubes, nanofibres, nanorods, NPs and thin films. The use of nanomaterials in biosensors permits the use of numerous new transduction innovations for investigations of different substances in vivo [9].

Other than biosensors, nanotechnologies additionally consider the use of biomolecules and procedures that rely on their use for the advancement of novel practical materials devices, nanomachines and maybe nanorobots. Generally, the latest cellular and sub-cellular nanodevices might be compelling in logical and medicinal applications, giving researchers and medical professionals one of the most powerful instruments possible to solve critical issues, treat human illness and overcome ageing-related health problems [10,11].

Considering the above points, several review papers have discussed the key aspects of nanomaterial design from different points of view. They include drug formulations for the treatment of specific populations (paediatric) [5], broad geographical-based assessments for nanotechnology [6], current status of the applications of nanorobotics in medicine [7], special applications (e.g., antioxidant) for nanomaterials in cancer treatment [8], implementations of nanomaterials in bioimaging technologies [9], nanoelectrodes in contact with skin [10], nanodevices for a sub-cellular basis [11] and others. However, there is a general lack of critical review that specifically disseminates the relevant knowledge for the design of NPs’ shapes and sizes and their synthesis methodologies and characterisation techniques for commercial biomedical applications, i.e., outside the typical small-scale laboratory experiments. We attribute this gap in discussion to numerous difficulties associated with the commercial-scale (bulk) production of nanomaterials for biomedical applications. Among these difficulties, one must figure out how to deliver cost-effective production of nanomaterials in large volumes, which needs one to define a preparation method that can be scaled up sufficiently to cover the cost required for targeting volume markets. Furthermore, supplying the nanomaterials in a specific structure for inclusion in the assembling form is not trivial, and it depends on the above-mentioned factors.

Interestingly, the behaviour of NPs’ surface and how these particles can be scattered in a wide assortment of media are critical, which can be controlled by adjusting the NPs’ design parameters. NPs’ size consistency and the unwavering quality of the chemical compositions are sometimes achievable for simple compounds such as oxides of NPs, but doing this for a mind-boggling number of materials in volume manufacturing is not trivial. Characterisation of nanomaterials is conceivable to portray materials to an extraordinary degree. A significant number of methods are appropriate for examination in the laboratory, but not for the production environment. Quick, mass and ideal techniques are required to screen properties such as particle size. All these together suggest an urgent need for the current review, as it introduces detailed information on these factors.

**Figure 1:**
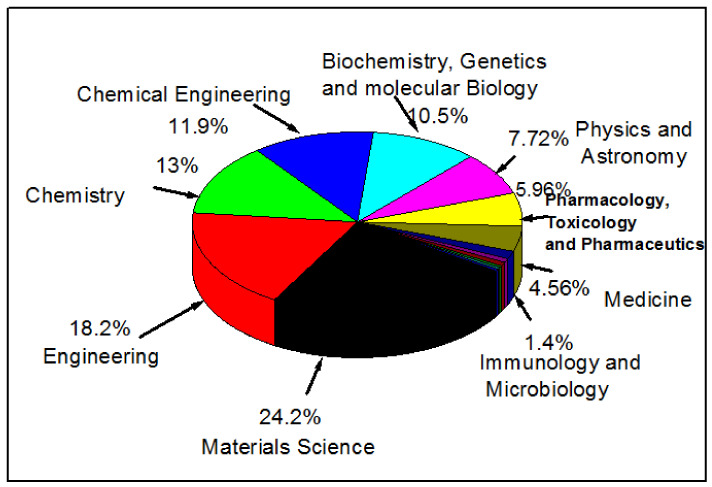
An analysis of the published documents according to their subject area in Scopus under a key word search on nanomaterials for biomedical and other applications such as engineering and materials science.

The above consideration is supported by the following analysis that we have carried out using data from Scopus for published articles dealing with the design of nanomaterials. This analysis has demonstrated a low percentage (about 12.5%) of publications relating to the applications of nanotechnology specifically in biomedical and biological engineering areas (Figure 1) as compared to other fields. Therefore, the current review article is aimed at supplementing this small body of work as well as filling the gap of a lack of analysis on recent trends and advances in nanotechnology for biomedical applications, including, but not limited to, tissue regeneration, medication delivery and wound healing. It should be noted that this review only covers non-biological materials for biomedical applications. To focus the discussion in the paper, we do not consider semimetals (or metalloids) and lanthanides that are used for medical applications sometimes.

## 2. Classification of Nanostructures

As discussed in the previous section, several classes of nanostructures have been reported in the literature, e.g., nanotubes, nanofibres, nanorods, etc. We extend this point further in this section by discussing the key aspects of different classes of these nanostructures. In particular, we attempt to define each nanostructure, its production method and the key factors (e.g., pH, use of surfactants, production temperature) that determine its production and applications. Where relevant, the most common methods for production of these nanostructures are also pointed out (e.g., mini-emulsion technique, seeding growth technique).

### 2.1. Nanospheres

**Figure 2:**
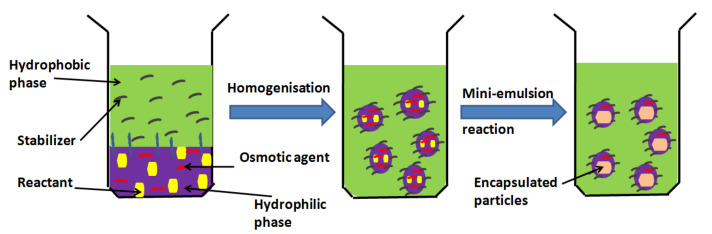
A schematic of a mini-emulsion process used for the preparation of encapsulated nanospheres (adapted from [13]).

Nanosphere innovation spins around the use of polymeric, inorganic or their combinational materials to produce spherical particles with average diameters in nanometres. Their nanoscale size and capacity to encapsulate and release drug or active molecules make them perfect vehicles for these purposes [12]. Nanospheres can be designed through implementation of a common technique such as the mini-emulsion technique (Figure 2), in which a precursor is dissolved into an organic solvent (oil phase). This is mixed with an aqueous solution of water and a surfactant or stabiliser. This is then homogenised further using a sonicator, and eventually, nanospheres are gathered by ultra-centrifugation [12]. The active molecules and medications may be loaded within the aqueous or the oil phase to guarantee their maximum fusion into the nanospheres. The morphology, size and drug encapsulation of the nanospheres can be characterised via different types of electron microscopy. Controlling the precursor concentration, precursor surfactant or stabiliser ratios and the homogeniser speed enables controlling the nanospheres’ diameter (size) and shape [13].

**Figure 3:**
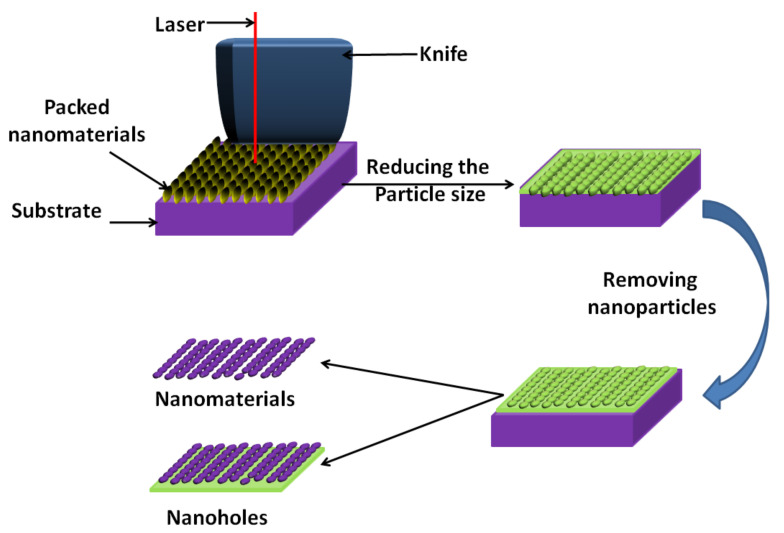
Schematic diagram for nanosphere lithography (adapted from [17]).

Nanosphere lithography (NSL) is also a common technique that is used for the preparation of nanosphere depositions on colloidal crystals. These facilitate the fabrication of various nanostructures on planar and non-planar substrates with low-cost, high-through put processing, a large fabrication area and a broad choice of materials. This strategy combines the benefits of both top-down and bottom-up methodologies. The procedure is divided into two stages. The first is to use ready-made, well-backed nanospheres of silica or latex as a substrate for the lithography process. The second stage includes substrates covered with a suspension containing monodisperse colloids (e.g., polystyrene) after a compound treatment to improve their hydrophilic character. After drying, a hexagonal close-pressed (HCP) monolayer or bilayer, called a colloidal crystal mask [14] (CCM), is framed, and this mask is then used to design new nanospheres (Figure 3). The subsequent removal of the mask (lift-off) by sonication in an adequate solvent or by stripping leaves an array of ordered nanodots on the surface of the substrate. An annealing step is sometimes necessary to crystallise the sample or/and induce a crystallographic phase change. Nanosphere lithography is otherwise called colloidal lithography or characteristic lithography [14,15].

In 1981, Fischer and Zingsheim [16] were among the first to report the arrangement of a well-baked monolayer on a glass plate. They deposited nanospheres in colloidal form with a particle diameter of >300 nm and allowed them to dry. During the 1990s, the technique was renamed by Hulteen and Van Duyne [17] with the name, as we know it today, nanosphere lithography. Hulteen and Van Duyne managed to develop a duple layer (DL) using this technique, which prompts the arrangement of little specks relating to the little openings that stay in a shut, pressed structure [17]. Thus, the plasmon reverberation properties of metallic materials were explored with a definitive objective of creating biosensors dependent on surface-enhanced Raman spectroscopy.

Furthermore, a few gatherings have hypothetically examined the behaviour of colloidal suspensions to comprehend their steadiness and additionally the system of arrangement of the mask [18]. Through the years, nanosphere lithography has been attracting attention because of its capability to fabricate a wide assortment of one-, two- or three-dimensional nanostructures [14,15,16,17,18]. For biomedical applications, the most reasonable strategy for manufacturing nanospheres by this method with desired properties ought to be accurately assessed. Most importantly, the materials included ought to be biocompatible and biodegradable. However, some of the limitations identified with the manufacturing strategies should be contemplated, including the designing goal, patterning resolution, fabrication cost, diversity of material selection, throughput, complexity and capability for large-scale production. In any case, the expenses of making a mould used in the creation of nanospheres drastically increase with the diminishing component size and the expanding area of the mould. In the long run, industrial production is a definitive objective of scientists. In this way, the capability of scale-up is an important factor to be considered while deciding the strategy to create nanoparticles (NPs) for biomedical applications. Regardless of the way that the above strategies have been embraced by the industries to fabricate different NPs, adjusting the condition for large-scale production is still very challenging.

### 2.2. Nanorods

Nanorods are NPs that possess dimensions below 100 nm, while the areas of two of the faces have an aspect ratio between 3 and 5 [19]. General properties of nanorods are significantly improved when compared to spherical particles. This is because the expansion of the aspect ratio prompts the expansion of excitation of surface plasmons in the NPs. Flexible medication delivery devices using nanoporous layers that comprise gold nanorods and dendrimers have been shown to give light-activated, on-request pulsatile discharge resources containing exceptionally enhanced therapeutics for a real patient’s recovery. The medication discharge rate is specifically associated with the temperature increment and lighted vitality of a near IR laser in both static and fluidic gadgets. This biocompatible stage for on-request control has been additionally affirmed by in vitro tests. Strikingly, unique reactions for improvements have been achieved from each medication in the excitation state in comparison to the non-excitation state, demonstrating the flexible capability of on-request slow medication delivery in special treatment regimes. These medication nanocarriers will enhance therapeutic efficacy and diminish side-effects due to the adjustments of plasma medication profiles [19]. Hyperthermia is a beneficial classical disease treatment, yet it is constrained by resistance of adjoining typical tissues. Parenteral organisation of gold nanorods (NRs) as a photosensitiser enhances the impacts of hyperthermia treatment, while saving typical tissues [20]. The essential factor in fabricating NPs to internalise a cell layer is their size (Table 1), which can be in a very wide range in nanometres [20]. The NPs enter the cells through active or passive pathways, yet their size can impact the mechanisms of internalisation, medication release and immune reaction [20]. For instance, in all pathways of receptor-mediated endocytosis, activation of targeted cell receptors using specific ligands on the surface of the NPs is responsible for initiation of membrane wrapping. Expanding ligand thickness on the nanoparticle surfaces requires a bigger size and a higher aspect ratio, which is the ratio of the length to the width of NPs. In the event that the diameter of spherical NPs is below 30 nm, they cannot drive the cell internalization. While those NPs over 60 nm can cause receptor saturation and strict hindrance to take place, the most optimum range of size is from 10 to 60 nm for higher cell uptake, without considering the surface charge and composition of the NPs [20]. However, utilising some bacterial species that produce carbohydrate secretions, which is a waste product, has appeared to assume a significant role in coordinating the molecular size and morphology of nanorods, alongside their production capacity. This can be promising as it presents cost-effective and sustainable alternatives in contrast to the conventional procedures.

### 2.3. Nanostars

Different metallic star-shaped nanostructures have been prepared for several biomedical applications [24,25]. In this case, the strategy involves producing a gold nanoparticle star (GNS) without the use of lethal surfactants generally required for GNS fabrication, which enhances the biocompatibility of the GNS. AGNS contains various sharp edges, which act like lightning poles to drastically enhance the nearby electromagnetic field. The structure results in more grounded surface-enhanced Raman scattering (SERS) improvement, increased two-photon activity cross-segment and higher photothermal transformation effectiveness as compared to other gold nanoparticle shapes [24]. Likewise, GNSs were used to examine nanoparticle infiltration through the blood–membrane barrier in cerebrum tumours [24]. Although different properties of GNSs have been researched, their in vivo biodistribution, tumour take-up, intra-tumoral dissemination and possibility of in vivo computed tomography (CT) imaging, surface-enhanced Raman scattering (SERS) location and tumour proliferating trichilemmal tumour (PTT) removal have not yet been completely examined. In this examination, in vivo studies showed the use of the multifunctional GNS test for both imaging and treatment. This was performed utilising two-photon luminescence (TPL) imaging, CT and radio-labelling to inspect how NP size and infusion portion influence their biodistribution and intra-tumoral conveyance. These outcomes affirm that the created multifunctional GNSs can be implemented for in vivo following at various goal scales and, in addition, for image-guided PTT for the treatment of malignant growth [25,26,27]. For production of Ag and Au, the adjustment in reaction conditions, for example, pH, will prompt a difference in balance conditions. For this situation, the more acidic the pH turns, the less the reduction reaction is supported [28]. Accordingly, by expanding the grouping of H^+^ (decreasing the pH value), the half-response reaction is encouraged. In addition, the decrease in reduction is adversely impacted, which results in a more serious level of aggregation. In addition, the amount of surfactant and its type, along with the reaction temperature, are thought to affect the stability and monodispersity of the obtained NPs [29]. Generally, in the market, gold NPs have been delivered in different sizes and shapes, including re-dispersible powders, silica coated, organic gold or sphere shaped. The gold NPs available in the market are relatively expensive; however, this is not considered as a challenge for the users, as the NPs are very effective in low doses.

### 2.4. Nano Core–Shells

**Figure 4:**
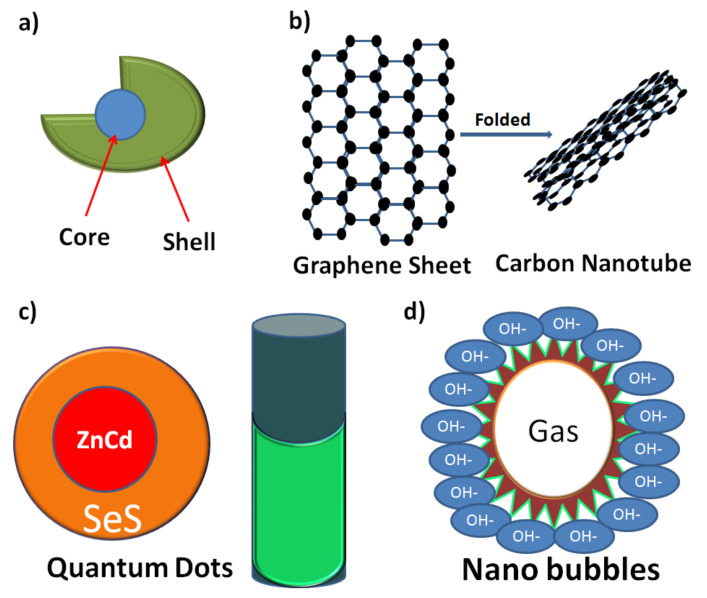
Demonstration of the configurations of (**a**) core–shell, (**b**) carbon nanotubes, (**c**) quantum dots and (**d**) nanobubbles.

Core–shell nanomaterials and nanostructures have turned into an essential research subject in the last couple of decades because of their potential applications in different fields, especially in the delivery of bioactive molecules, etc. (see Figure 4a) [30,31,32]. Core–shell nanocomposites and nanostructures might possess various sizes and distinctive states of core and shell thickness, with different surface morphologies. They might be spheres, cubes, rods, star-like or cylindrical shaped like a fiddle. The size and shape can be controlled based on the material properties. Singular core–shell NPs have different applications in various fields of therapeutic biotechnology, diagnosis, tumour treatment and drug delivery. Several methods have been used to change the surface properties of the NPs, including practical conjugation or thin-layer deposition with different materials (with various constituents). Functionalised NPs exhibit upgraded properties contrasted with the non-functionalised uncoated particles. There are distinctive core–shell structures, including (1) metalcore and different metal shell, (2) metalcore and non-metallic shell, (3) metalcore and polymer shell, (4) non-metallic core and non-metallic shell, (5) polymer core and non-metallic shell and (6) polymer core and polymer shell using different two polymers. For these six classes, the core and shell materials may be switched to tune different properties [27,32].

**Figure 5:**
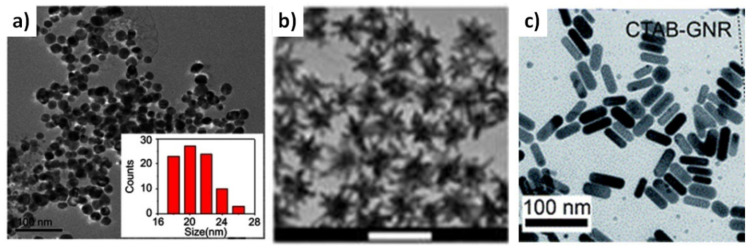
Representative transmission electron microscopy (TEM) images of (**a**) silver nanospheres [22], (**b**) gold nanostars [25] and (**c**) gold nanorods [19].

A critical job in this regard can be achieved using core–shell NPs in biomedical applications. They are considered as medication carriers with controlled release. By changing the orientation and morphology of the centre and shell materials, loading and release performance can be adjusted. For example, self-assembled 9Ps Fe_3_O_4_ @mSiO_2_core–shell nanocrystals produced in the absence of a surfactant were loaded with hydrophobic medication particles within the NPs’ centre that afterwards was covered with a thin shell of SiO_2_ [33]. When the medication is loaded in the core structure, prolonged release is the target, and for quick burst release, the medication is to be loaded in the outer layer (shell). In other words, the slower and sustained drug release performance demonstrated straight and controlled discharge for the active ingredients loaded within the core matrix. These core–shell nanocarriers likewise demonstrated high cell infiltration [33,34]. Core–shell designs enhance the beneficial applications of NPs in the industry of targeted anti-tumour drugs, as they can deliver the active ingredients slowly upon its localisation into the cancer tissue, thus, guaranteeing minimal side effects on the normal tissues and greater effects on the cancerous tissues. This is a solution for hypoxia-induced reactive resistance of tumour cells because of lower anticancer delivery with systematic therapies. Some specific examples of nanospheres, nanostars and nanorods are discussed in Section 2.8 and illustrated in Figure 5.

### 2.5. Nanotubes

Among nanotubes, carbon nanotubes, which were invented in 1991, are the most well-known [35]. They are cylindrical structures like a sheet of graphite folded into a chamber topped at one or both ends, finished by a buck ball (see Figure 4b). Due to their structures, nanotubes can be classified into two types: single-walled carbon nanotubes (SWCNTs) and multiwalled carbon nanotubes (MWCNTs). The inner width of the former ranges between 1 and 2 nm, and the latter has an inner width range of 2–25 nm with 0.36 nm separation between each layer. Their variable length ranging from 1 μm to a couple of micrometres [36] enables them to be used in the field of biomedical applications, especially as medicine carriers. In addition, their characteristics enable scientists to attach some antibodies accompanied with radio- or fluorescent labels to their surface for cell-level diagnoses or treatment [37]. Cell internalisation of nanotubes is through the outer cell membrane by endocytosis. Nanotubes can be used as a transporting system for bioactive molecules such as medications or genes after their functionalisation with ammonium or carboxylic groups to get them into a soluble form [38].

Nanotubes have shown enhanced medication conveyance of amphotericin B to the inside of cells compared to drug delivery without nanotubes. The viability of amphotericin B nanotubes is more noteworthy as an antifungal operator contrasted with amphotericin B alone, and they are effective on strains of parasites that are typically impervious to amphotericin B alone. Further, there is decreased cell cytotoxicity towards mammalian cells treated with amphotericin-B-loaded nanotubes [39]. The capability of nanotubes to transport DNA transversely over a cell layer is used in cell cytotoxicity studies. DNA can be joined to the tips of nanotubes or can be merged inside their chambers. Prato et al. [38] provided an increasingly imperative explanation of the β-galactosidase marker quality through nanotubes appearing different compared to uncovered DNA. This shows the advantage of the non-immunogenicity method over viral vectors used for cancer diagnosis and treatment. In addition, it offers an empirical look at small interfering RNA (siRNA) developed as an approach to cancer treatment where tumour cells are explicitly changed. Functionalised single-walled carbon nanotubes with siRNA were used in this regard [40].

It was demonstrated that carbon nanotubes (excluding acetylated nanotubes) when reinforced with a peptide create a higher immunological reaction compared to free peptides. This property can be used in antibody creation to improve the viability of immunisations. Water-insoluble nanotubes, such as unblemished carbon nanotubes, have high in vitro cytotoxicity compared with changed water-dispersible nanotubes. It was likewise observed that with functionalisation of water-insoluble nanotubes, the potential of cytotoxicity decreases. Moreover, functionalisation influences the clearance of the nanotubes. SWCNTs without conjugation to a monoclonal antibody have high renal and liver uptake when compared with monoclonal-antibody-conjugated SWCNTs [41]. During the past decade, it has been observed that commercial activities related to CNTs have substantially grown. The number of patents and journal publications accompanied with a large production of CNTs is annually increasing. On a worldwide basis, it is predicted that the production capacity of these nanomaterials will be about 7 tonnes of SWCNTs and 300 tonnes of MWCNTs, owing to the high demands of about 1000 tonnes/year. It is worth highlighting that the vertical design of the furnace used is ordinarily implemented in the large-scale manufacturing of carbon nanotubes.

### 2.6. Quantum Dots

For both biomedical and modern applications, quantum dots (QDs) are among the most developed entities of NPs widely used in the last decade. The idea of QDs was created in strong glass crystals and a fluid state. According to quantum mechanics, QDs can be defined as any materials that possess higher electrical and optical properties in their nanoscale (2–10 nm) compared to their largescale, which makes them highly recommended for semiconductor applications. This explains the way that the optical properties of nanocrystals (NCs), for example, dots, originate from quantum mechanics. It is thought that QDs will form a new future of semiconductor inorganic crystals [42]. Fundamentally, QDs comprise a semiconductor centre, covered by a shell, and a cap which enhance their dissolvability in watery solutions (see, Figure 4c) [43]. QDs are core–shell structures in the nanoscale; the core is made of an inorganic material and responsible for semiconducting properties and optical characters. On the other hand, the outer layer is composed of organic surfactants attached to ligands.

QDs can be used for biomedical purposes as a diagnostic and curing device. They can be labelled with biomolecules and used in exceedingly delicate tests. An examination conducted on prostate malignant growth created in naked mice demonstrated aggregation of QDs [44]. QDs can likewise be used for imaging of tumours in malignancy patients for tumour mapping and managing suitable treatments. This strategy can be conducted for different cancers, including melanoma and bosom, lung and gastrointestinal tumours [45]. They are appropriate contenders for the diagnosis of cells and model animals owing to their exceptional characteristics, and they might replace the organic pigments used originally for the same porous materials due to their known disadvantages as well as limitations [46]. The use of QDs in a clinical setting has restrictions attributable to their end products. Functionalisation of QDs, which shields tissues from their toxic side effects, prompts a more prominent increase in the size of nanoparticles than the pore size of the endothelium and renal vessels, hence lessening its end and causing harm. Additionally, there is no information about the clearance and digestion of QDs in in vivo studies [46,47,48]. Some QDs are illustrated in Table 2, along with their emission ranges and sizes. On a practical basis, the charge of the shell that traps the core may reduce the fluorescence of the fabricated QDs. A hole and an excited electron, either both or one of them, may also migrate to the QDs’ surface. Moreover, if the hole or the excited electron is absorbed, it will decrease the time of decay and thus negatively affects the fluorescence of the fabricated QDs. These limitations hinder the applicability of QDs in a sustainable manner. There is little known about companies who provide the market with QDs, and they are only for research purposes now. This could be due to the significant expense that confines their use to profoundly particular applications. As anticipated by industry experts, this could change over the next years, since increasingly proficient assembling forms are right now being searched for by researchers.

### 2.7. Nanobubbles

Medications for diseases can be formulated into nanoscaled bubble-like structures called nanobubbles (see Figure 4d). The mechanism by which nanobubbles deliver drugs is that the body temperature makes them unstable and makes them aggregate to form microbubbles using external ultrasonic stimulation, resulting in the release of the active ingredients encapsulated in the nanobubbles. Their main uses are in the treatment of tumour tissue and conveying the medications, specifically those affected by ultrasound stimulation. Nanobubbles can skip normal cells and expand the intracellular internalisation of the medication by the tumour cells. Likewise, they have an extra advantage of allowing imaging of the tumour by ultrasound techniques [49]. A successful trial for nanobubbles being used as carriers for doxorubicin as an anticancer drug on the basis of in vitro and in vivo studies was reported by Rapaport et al. [50]. They used an ultrasound external source to cause disturbances for the aggregated nanobubbles that in turn started the doxorubicin release. 

The aggregated nanobubbles held the medication in a steady state until invigorated by high-intensity focused ultrasound (HIFU).This, in turn, elevated the amounts of medication in the target cells and subsequently decreased mortality and increased viability. This strategy needs further investigation for its utility in the treatment of different malignancies. Other applications of nanobubbles apart from cancer treatment include their use as a carrier for non-viral vectors for the treatment of some diseases [51] or the diagnosis of some diseases with the help of ultrasound imaging [52,53], and they are additionally being tried as a remedial measure for clot removal in a vascular framework combined with ultrasound. This technique has focal points of being non-intrusive, making it harm the endothelium less [53]. Nanobubbles exist already in the market of biomedical materials, and several pharmaceutical companies provide an ultrasound contrast agent (UCA) that is loaded with microbubbles/nanobubbles. Physicians usually inject a UCA in the patient’s blood to enhance its acoustic scattering, which helps in visualisation of non-reachable organs such as the heart.

### 2.8. Nanocrystal and Nanocube Structures

Changing metallic or bimetallic (BM) NPs, which start with one shape and then change onto another desired shape, is of significance to nanoscience and nanotechnology. This is because new morphologies of NPs lead to improvement of their exploitable properties. Recently, colloidal inorganic nanocrystals (NCs) have received extensive consideration in a few fields according to their fascinating size-subordinate properties, for example, their quantum control effect and restricted surface plasmonic effect, which are not recognised in their bulk status [54,55]. The crystallinity of metal-oxide NPs significantly affects their properties and is regularly showed through various reactivities relying upon translucent directions of their surfaces. Among the nanocrystals, nanocube structures have gained great attention in the field of nanosensors. For example, in the light of the localised surface plasmon resonance (LSPR) innovation, a novel plasmonic nanosensor with high affectability and high selectivity was recently fabricated for the recognition of trace sulphide particles on an Au@Ag nanoparticle [56,57,58]. Moreover, the seeding growth method has been considered as one of the successful techniques for obtaining nanospheres, nanostars and nanorods during the past decades. The seeding growth method has been demonstrated to be excellent with regard to controlling the size of NPs [59]. In this method, small metal NPs are first synthesised and used as seeds (sites of nucleation), which are blended in with a growth solution for the preparation of bigger particles. The obtained seeds and growth solution are able to restrain further nucleation, which prompts a non-homogeneous molecule size in the stable colloidal solution with the stabilising agent’s assistance [60]. For instance, different substrates were used for the preparation of ZnO nanostructures with controlled morphologies, where it was shown that a seed layer of 100 nm was helpful in achieving well-aligned nanorods of ZnO [61]. It was also reported that by changing the seeding metal morphology, the final structures of heterogeneous metal-TiO_2_ nanomaterials and their applications could be controlled [62].

## 3. Production Methods of Nanomaterials

### 3.1. Approaches for the Preparation of Nanomaterials

**Figure 6:**
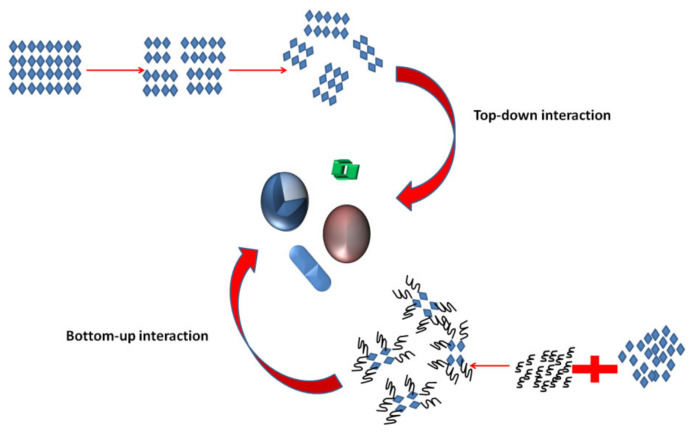
Diagram demonstrating the difference between top-down and bottom-up theories (adapted from [63]).

There are two types of approaches for the synthesis of nanomaterials and the fabrication of nanostructures. Top-down approaches refer to slicing or successive cutting of a bulk material to nano-size particles, which can be achieved through attrition and milling. Bottom-up approaches refer to methods where materials create themselves by self-assembly. Chemical reactions are good examples of this technique (see Figure 6). The bottom-up approach is most common in the production of devices in parallel, and it is a cost-effective method when compared to top-down strategies; however, overseeing the techniques is troublesome when things turn out to be bigger and more cumbersome than what is regularly made by substance union [63]. Most of the biomedical supplies in the market are part-manufactured through these strategies, for example, moulding, carving and cutting. Owing to the impediments in these procedures, significantly advanced nanotools are yet to be made.

#### 3.1.1. Inorganic NPs

Inorganic NPs are very common in the pharmaceutical industry as they can provide alternatives for traditional antibiotics and anti-cancer drugs, thus creating new impetuses for their usage in this field. These new impetuses have invigorated the enthusiasm of pharmaceutical organisations associated with the improvement of non-conventional medications, particularly nanotechnology enterprises, which have put resources into the improvement of new nanomaterials recognised as promising operators against microbes impervious to conventional antibiotics and anti-cancer drugs.

##### Chemical Precipitation

The chemical precipitation method is very common in the fabrication of different nanomaterials for biomedical applications owing to its simplicity and inexpensive starting materials. In this method, the basic challenges are to avoid the physical changes in and the aggregation of tiny crystallites that might develop during the synthesis procedure in the preparation medium. For this purpose, non-aqueous solvents at lower temperatures are employed to induce double-layer repulsion to control thermal coagulation and Oswald ripening. The reaction in this method takes place in a suitable solvent for all the reactants, and the dopant materials should be included prior to the precipitation. In addition, to prevent the NPs’ agglomeration, some surfactants can be applied. Finally, the NPs can be further collected and washed using a centrifugation instrument and dried in an oven. Sometimes, it is also useful to use UV to polymerise the surfactant layer that caps the NPs to maintain their original sizes [64,65]. The previous literature has highlighted the effects of the surfactant types on the final characteristics of NPs and their influence in controlling their applications [66,67].

##### Sol-Gel Technique

The sol-gel processing technique has been extensively used for the preparation of NPs for biomedical applications. As is well-known, NPs or regular molecules are smaller than colloidal particles, and in the case of their dispersion in aqueous solvents, they form clear solutions, unlike colloidal particles, which form a bulky solution. The main principle of this process is the formation of a continuous liquid-phase network (gel) from the colloidal suspensions of the starting materials (sol). These starting materials are complexes of metal alkoxides ions and aloxysilanes. The following alkoxides are the most highly used starting materials for the sol-gel technique: tetramethoxysilane (TMOS) and tetraethoxysilanes (TEOS), which form silica gels owing to alkoxides’ immiscibility in water. They are organometallic precursors for silica, aluminium, titanium, zirconium and many others, and alcohols are the mutual solvents used in this technique. At least one or more of the chosen alkoxides should be included in the sol-gel technique to obtain homogeneous nanomaterials [68,69,70,71,72]. In practice, the chemical precipitation technique shows greater superiority if compared with the sol-gel technique as it requires low-cost starting materials and facile and simple preparation steps, along with easy scale-up manufacturing techniques.

#### 3.1.2. Nanopolymers

**Figure 7:**
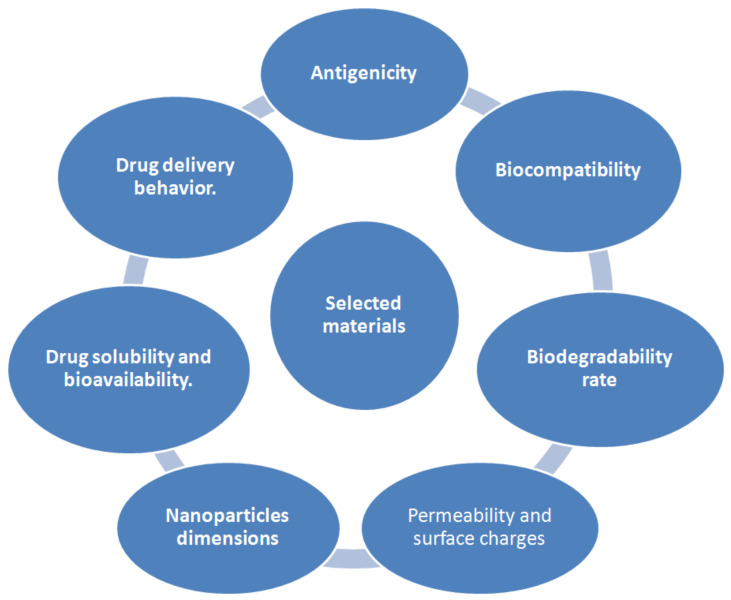
Key elements that should be considered while selecting nanomaterials for biomedical field.

The decision of selecting a reasonable procedure for the formation of NPs depends upon the physicochemical characteristics of both the polymer and the drug to be used. NPs can be designed from a variety of materials, including proteins, polysaccharides and manufactured polymers. The choice of grid materials is also subject to numerous elements that are summarised in Figure 7 [73].

Three techniques were used for the designing of NPs, as previously reported, polymer dispersion, ionic gelation of hydrophilic polymers and non-wetting polymerisation of monomers. In the non-wetting polymerisation method, hydrophobic monomer molecules and free-radical initiators are added to a bath of a water-based emulsion alongside surfactants. The surfactant molecules are made of hydrophilic and hydrophobic ends, which are responsible for balancing out the emulsion before polymerisation by covering the monomer beads. Other surfactant molecules cluster together into more modest totals called micelles, which additionally assimilate monomer particles. Polymerisation takes place when initiators relocate into the micelles, instigating the monomer molecules to frame large molecules that form a polymer. Non-wetting polymerisation enables adjustment of the shape, size and composition of the fabricated NPs and is capable of large mass production, which is why this method is widely used in the industrial field. Successful production of polymer nanomaterials has been achieved for several polymers, namely common biodegradable polymers such as poly(D,L-lactide-co-glycolide) (PLGA), poly(D,L-glycolide) (PLG), poly(cyanoacrylate) (PCA) and poly(lactic acid) (PLA) [74,75,76]. Some physicochemical features that can be used to screen the characteristics of polymeric colloidal suspensions are particle diameter, surface charge, polymer molar mass dissemination, loaded medication and pH. It was reported that by adjusting the pH of the solution, a star polymer can self-assemble into nanostructures via controlled host–guest interactions between the cyclodextrin polymer and hydrophobic guest molecules [77]. However, the modern use of polymeric NPs can be restricted because of issues of low physicochemical stability during a long period of storage. The principal impediments are the aggregation of particles, chemical stability of the polymer, the medication or other crude materials used during NP synthesis and, furthermore, the untimely delivery of active molecules. Despite everything, this field needs much research since numerous issues remain uncertain. It is hard to control the particle diameter/thickness and morphology during the creation of polymeric nanomaterials. Besides, the way by which an error is detected in polymeric nanomaterials has not been completely figured out. Finally, specific development components have not been completely comprehended, and the job of variables in deciding the morphology of, for example, temperature, heating rates, ionic quality and dissolvable consistency is very critical. A low synthesis temperature is always recommended for the preparation of stable polymeric nanoparticles, especially for the applications of bioactive composites [78]. In addition, organic ligand attachment to polymeric NPs is just hypothetically described. Above all, functioning, cytotoxicity and the body clearance related to the different engineered courses of these materials should be expeditiously managed for their general acknowledgment and appropriateness, practically speaking.

#### 3.1.3. Nanocomposites

Nanocomposites are multiphase materials in which one or more of the material particle size is below 100 nm. The features of nanocomposite materials depend not just on their native individual molecules/particles but also on their morphology and interfacial behaviour. Most nanocomposites, which have demonstrated innovative significance, are made of two materials. Nanocomposite-based polymers have been extensively used in the pharmaceutical industry; in fact, most of the pharmaceutical formulations on the market (especially those related to wound healing or skin regeneration) are made of hydrogels composed of biopolymers loaded with either nano-metal oxides or other active ingredients. Therefore, it is suggested that the scaling up of nanocomposites in the biomedical industry will be doubled soon to include all market requirements. For instance, it has been proved that by deposition/mixing metallic ions on/within a polymer matrix, nanocomposites based on polymers are achievable through photolithography and electrospinning techniques [79,80,81,82,83,84].

##### Photolithography

Lithography is now considered as an intelligent and innovative system for preparing monodispersed particles, although it was used only for usual printing in the early stages [79]. The way it works is very simple: a raised surface is covered with ink, and a copy of this surface is recorded by applying paper or a material in contact upon the inked surface. The same technique is also applied in advanced lithography that depends on imprints engraved onto a plate to hold the ink [80]. The process depends on the difficult mixing of oily materials with an aqueous one. Structures are drawn or painted with an oil-based substance (oily ink) on exceptionally arranged limestone. The limestone is saturated with water, which the limestone acknowledges in zones not secured by the pastel. A slick ink, connected with a roller, clings just to the illustration and is repulsed by the wet parts of the limestone. The print is then made by squeezing paper or materials against the inked illustration. Photolithography is a strategy used to exchange shapes and structures onto a surface of photoresist materials.

Through the years, this procedure has been refined and scaled down. Microlithography is now used to deliver objects, for example semiconductors for PCs and a variety of various biosensors. To date, photolithography has stood out amongst the best advancements in the field of micro-fabrication [81]. Successful examples of the use of this technique in biomedical applications have been reported [80,81]. A glucose biosensor was fabricated using photolithography, and for this purpose, a silicon wafer was spin-coated with AZ-1518 as a photoresist. This was further coated with a hexagonal close-packed circle array to develop a hexagonal close-packed column array of AZ-This biosensor was found to be very efficient in detecting non-enzymatic glucose in a linear range [80]. The production of shape-defined proteins is also a successful example of photolithography as a designing method for proteins, as it is applicable for different biomedical applications such as medication transfer and biochemical detectors [81].

##### Electrospinning (Originated from Electrospraying)

**Figure 8:**
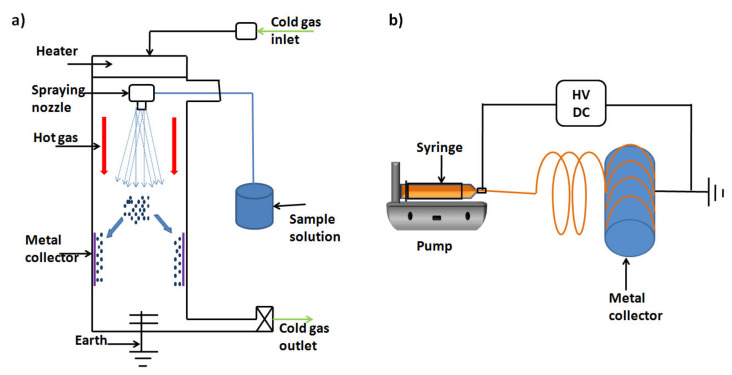
Schematic diagrams of (**a**) spray dryer and (**b**) electrospinner.

To fabricate nanofibrous materials, there are a few methods that can be applied. Among these techniques, electrospinning has gained the highest share, especially for biomedical applications and tissue engineering. It can be defined as a strategy used to make polymeric fibres with widths in the nanometre scale. This procedure includes the spinning of a charged solution of a polymer onto an oppositely charged surface (collector). One of the primary examinations concerning the flowability of the solution between the collector and the nozzle tip was controlled by adjusting the distance between them [82]. A lot of these investigations used watery electrolyte arrangements with high electrical conductivity and low thickness. The expansion of a charge to this arrangement made it frame fine splashes of charged beads that were pulled into the counter-cathode. These beads immediately dissipated noticeably all around; this procedure was later called electrospraying (see Figure 8a). Electrospinning is fundamentally the same as electrospraying; a charge is connected to a polymer arrangement and launched out towards an oppositely charged target (see Figure 8b). In the two procedures, the surface strain and viscoelastic strengths of the polymer arrangement cause drops at the tip of the syringe to hold their hemispherical shape. The charge actuated by the electric field makes the bead disfigure into a Taylor cone at the tip of the cylinder [83]. At the point when the connected voltage is expanded above the critical limit, the electric power in the bead overcomes the contradicting surface tension and a very tiny fibre is launched from the tip of the Taylor cone.

In electrospraying, the quality of the electric field and low solution viscosity causes beads to isolate from the cone and splash onto the objective. In electrospinning, the high voltage applied on the polymer causes a fibre to be expelled from the tip of the cone. Many successful trials were conducted using electrospinning technology to produce nanofibres with intelligent properties in the fields of biomaterials and drug delivery systems. To mention a few, Mabrouk et al. [84] developed calcium silicate/Poly(vinyl) alcohol (PVA) nanofibre wound-dressing material using a nano-electrospinning device. This wound dressing proved its bioactivity and antibacterial properties in vitro. In addition, a nanomembranous patch for the treatment of diabetic foot ulcers loaded with the broad-spectrum antibiotic ciprofloxacin was also reported [85]. This paper’s co-author Das’s research team modelled a membrane bioreactor where membranes developed by the electrospinning technique may be used. His team has also conducted laboratory experiments to investigate solute diffusion through these membranes in different mediums, including water and cell culture medium [86,87,88].

## 4. Characterisation of NPs

**Figure 9:**
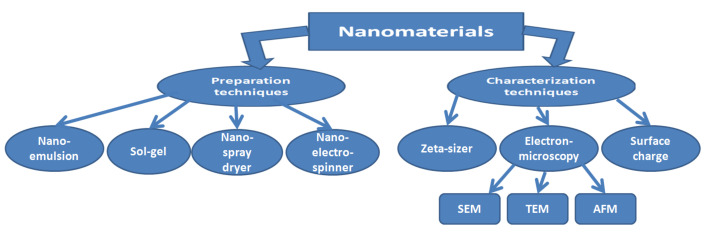
Illustration of common techniques for preparation and characterisation of nanomaterials.

The particle size distribution, diameter and shape are very necessary parameters to evaluate the designed NPs and can be determined by several characterisation techniques. These techniques include a zetasizer, scanning electron microscopy (SEM), transmission electron microscopy (TEM) and atomic force microscopy (AFM), especially for the particle diameter, shape and phase of dispersion. Common preparation and characterisation techniques are illustrated in Figure 9.

### 4.1. Dynamic Light Scattering

**Figure 10:**
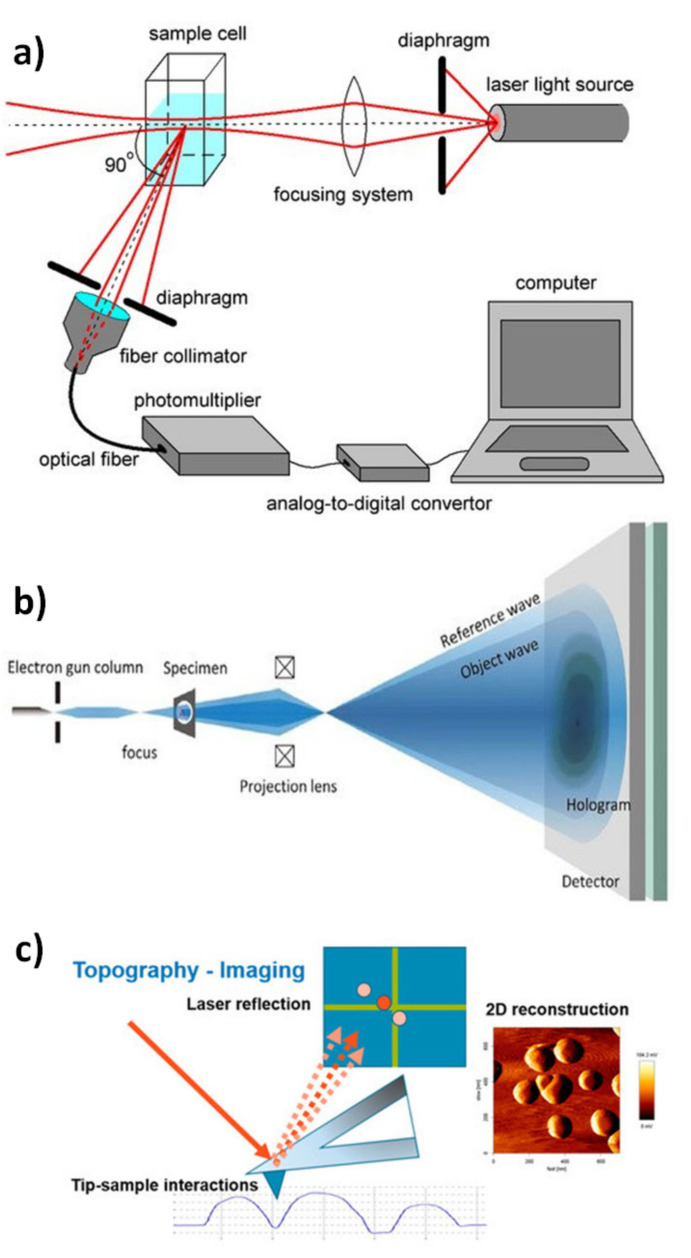
Schematic representations of (**a**) dynamic light scattering (DLS) [89], (**b**) transmission electron microscopy (TEM) [90] and (**c**) atomic force microscopy (AFM) [91].

Dynamic light scattering (DLS) is considered as one of the fastest techniques that are still used for NP characterisation and most favoured by scientists who are interested in NP design. The technique can be applied only for NPs that can be suspended in a colloidal solution and induce a Brownian motion in the solution. The DLS technique relies on the detection of the Doppler shift that is caused by the NPs when subjected to monochromatic light (laser) (see Figure 10a). This, in turn, causes a shift in the detected light, and the degree of this shift determines the size of the investigated NPs. In addition, several parameters can be obtained from this technique, such as the particle diffusion coefficient by using the autocorrelation function. When it comes to evaluating the distribution of particles, DLS is the most prevalent technique for this purpose [89,90].

### 4.2. Scanning Electron Microscopy

Among the available microscopic techniques used for determining NPs’ shape, surface characteristics and diameter, scanning electron microscopy (SEM) determines these important features. Limited data on the size distribution and true population average are provided by this technique, and only dry powder is applicable, while liquids are prohibited. Sample preparation requires fixing of a sample powder on the surface of special holders (stubs), and this is followed by coating with an electron-conducting material such as gold or carbon for a sufficient amount of time [89]. The specimen surface characteristics are investigated by detecting the secondary electrons discharged from the sample surface. The particle size detected by SEM should be consistent with the results obtained for the same sample using a DLS zetasizer. However, these procedures are tedious and expensive and often require complementary data on size distribution [89].

### 4.3. Transmission Electron Microscopy

Employing traditional techniques for detection of the size and shape of NPs is very limited due to their tiny sizes. Impressively, optional information about the designed nanomaterials is obtainable using transmission electron microscopy (TEM) techniques, including spectroscopic data, image recording and diffraction. These can take place simultaneously or individually with variable resolutions (see Figure 10b). Although TEM provides the same information as SEM, they operate on a different basis. The specimen manipulation for TEM is intricate and tedious on account of its necessity to be ultrathin for electron transmittance. In the principal investigations of significance to nanoscience and nanotechnology, nanometre-resolution X-ray energy-dispersive, nanodiffraction and atomic-resolution electron energy-loss spectroscopy techniques with high-resolution TEM imaging are very beneficial.

Support films or grids are used to deposit a liquid-dispersed sample on their surface prior to testing by TEM. Furthermore, to hold the NPs tightly on these specific holders against the machine’s high vacuum, they must be covered with a thin layer of plastic film or stained with specific pigments; otherwise, liquid nitrogen can be applied to the specimen to freeze it completely. Eventually, as the electron beam passes through the specimen, the features of the sample are recorded [90].

### 4.4. Atomic Force Microscopy

This procedure is also called scanning force microscopy (a strategy that shapes pictures of surfaces using a probe that scans a sample). When compared with normal light microscopy, atomic force microscopy(AFM) offers 100 times’ high detailed resolution infractions of nanometres. The principle by which the image procedure is governed is that the sample is physically assessed by using a cantilever probe at the sub-micron level that results in a better-quality image as well as higher magnification, besides recording the particles’ topography and size (see Figure 10c) [91].

Two different image-recording modes are available in AFM, contact and non-contacting modes; only the specimen nature decides the applicable mode. Simply, contact mode means that the probe must touch the specimen surface, and non-contacting mode means the probe must not touch the specimen surface. Non-contacting mode allows this imaging technique to be used with very delicate specimens, especially those contain biological compartments [92,93]. In addition, AFM (with no further calculations) gives the most exact depiction of size, size distribution and actual image, which assists in understanding the impact of different biological conditions [94].

### 4.5. Zeta Potential

The material–cell interactions are greatly affected by the NPs’ surface charge and intensity; therefore, it is necessary to determine this property. Among the available techniques that can be used to assess the charge of NPs is the zeta potential technique that indirectly evaluates the surface charge. Its idea depends on calculating the differences between the NPs’ surface shear and the external Helmholtz plane. In this manner, the zeta potential of a colloid-based dispersion aids in specifically assessing its storage stability. Zeta potential values (high zeta potential values, either positive or negative) are achieved to ensure stability and avoid aggregation of particles. Zeta potential values can be used for assessing the surface hydrophobicity and material behaviour of the molecules incorporated inside the NPs or deposited on their surface [94].

## 5. Nanomaterials for Biomedical Applications

**Figure 11:**
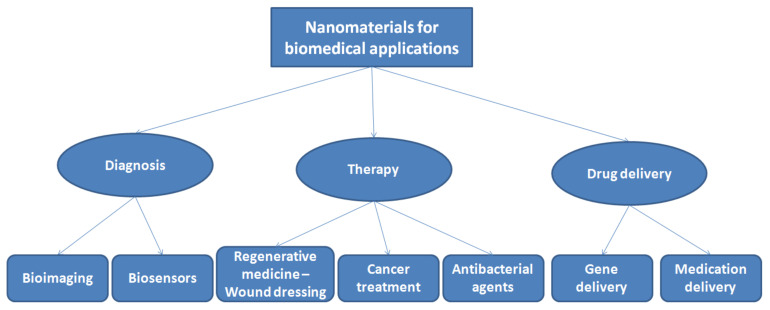
Common biomedical applications recorded for nanomaterials.

As the human population ages and the future expands, tissue wounds and pathophysiology will keep on expanding, imposing a real physical and money-related strain on the overall social insurance frameworks. To this end, it is foreseen that biomaterial NPs will offer the best way to deal with regenerative medicine that will assume an urgent role in the regeneration of damaged body parts. It is believed that the field of bioactive nanomaterials will keep on exponentially developing in the future, given the examples of overcoming limitations of biomaterial approaches in scholastic, clinical and mechanical-based procedures. The US market is expected to show increased expenses for bioactive nanomaterial supplies from USD 70.03 billion to USD 130.17 billion before the end of 2021, with a growth rate of 13.2%. Nanomaterials that can be classified as bioactive nanomaterials are divided into two categories according to their origin, either natural or synthetic nanomaterials; the general applications of nanomaterials in the medical field are summarised in Figure 11.

Their applications in the human body are governed by their bioactivity, mechanical, degradation and biocompatibility properties. Till now, several applications of biomaterials in biomedical applications have been already approved, such as heart valves [93], plastic surgery [95], joint substitutions [96], drug delivery devices [97] and other restorative applications [98]. The entry of new items in these fields, for example, plastic medical procedures, nervous system regeneration and wound treatment, is relied upon to enhance the development of this market. The market of cardiovascular biomaterials overwhelms other biomaterial markets because of high incidence rates of cardiovascular diseases, and the market of orthopaedic biomaterials is the second biggest market in the biomaterials field. There is a mismatch between the rate of innovation of nanomaterials and their application in the biomedical field. While most individuals from the exploiter network can get a handle on the significance of nanotechnology and can expertly dispatch and deal with a reasonable item on the market, they are restricted in their applied comprehension of this logical order and the unpredictable internal activities behind the item’s usefulness [94]. Those engaged in scientific research perceive that nanomedicine is a development of nanotechnology, yet their business mastery required to advance an item in business is very low [99]. Participation is hence required between the two groups in a quest to lead nanomedicine-based innovations to an effective showcase position. In general, biomedical applications include regenerative medicine as well as the therapeutic use of antibacterial and anticancer agents. However, many review articles have already focused on the applications of antibacterial and anticancer agents [100,101,102,103], and therefore, this review stressed on regenerative medicine, which, we believe, needs more extensive research.

### 5.1. Regenerative Medicine

Tissue malfunctioning has become a noteworthy medical issue in the United States and most other geographical areas around the world. It was reported that only the United States has around 89000 patients who need tissue or organ transplantation [99]. Recently, a brilliant interdisciplinary field was able to solve the issue of tissue and organ replacement, which is called nanomedicine. Nanomedicine provides nanomaterials that can be used as a substrate for cell growth and proliferation and can deliver medicine and biological molecules, which eventually results in new tissue or organ replacing the damaged body part under controlled conditions [104]. Among the various nanomedicine techniques is the guided regeneration technique, where synthetic biodegradable nanoplatforms like poly(L-lactic acid) (PLLA), polyglycolic acid (PGA), poly(D,L-lactide-co-glycolide) (PLGA), polycaprolactone (PCL), polyphosphazenes, etc., are used for directing cell growth [105,106,107,108,109].

Moreover, nerve generation is one of the most significant issues in regenerative medicine. Till now, it is a significant challenge to recover nerve tissue at injury sites. There are different types of nanomaterials that have been created and are under study to prevent or treat nerve damage. Many of the nanomaterials are promising, with appropriate physicochemical properties, and have been subsequently used for neural tissue regeneration applications. These have shown promising outcomes that can help cell attachment and expansion, advance neuronal cell separation and upgrade the recovery of neurons [109]. Some inorganic materials have been used in the regeneration of damaged nerves, such as metallic NPs [108], silica NPs [109], magnetic NPs [110] and quantum dots [111]. On the other hand, distinctive organic nanomaterials have been studied for neural tissue regeneration applications [112]. Polymeric NPs [113], liposomes [114], dendrimers [115], micelles [116], nanofibres [117] and carbon-based nanomaterials [118] are some natural nanomaterials.

In addition, the use of nanomaterials may be an appropriate solution since these materials can mimic the surface properties of damaged tissue. Consequently, in the past decades, nanomaterials have been featured as promising candidates for improving conventional tissue regeneration materials. Critically, these endeavours have shown that nanomaterials display unrivalled cyto-compatible, electrical, mechanical, synergist, optical and attractive properties compared with traditional materials. These impressive properties of nanomaterials have assisted in improving different tissue developments over what is reachable today. Nanomaterials have been proved useful for bone [119], ligament [120], vascular [121] and bladder tissue [122] regeneration applications as well.

Moreover, it is worth highlighting that wound treatments that adopt regenerative medicine approaches are becoming very popular. Humans are subjected to different wound impacts that may result from several causes. If this is to be defined on a biological basis, it would be when a person is injured. However, four major steps are involved in the evolution of a wound: haemostasis, inflammation, proliferation and maturation [123,124,125,126]. In the treatment of wounds, many approaches were developed during the last five decades. Among these approaches, nanotechnology had gained great attention among the scientists interested in this field. In addition, great preference was given to the inclusion of inorganic agents in these medication devices as antibacterial agents, such as Zn, Ag, Cu and others; loading of antibiotics in nanomaterials was also explored [126]. The usage of these NPs loaded with antibacterial agents or antibiotics is demonstrated to be the best aid in the treatment of wounds. Nanofibres loaded with bioactive molecules, drugs and antibacterial agents are considered as one type of polymer nanomaterials. They have exhibited incredible results when used as skin regeneration materials or patches [84,85].

Electrospinning has been generally used as a nanofibre manufacturing method. Its basic procedure, cost viability and adaptability have been recognised by materials researchers universally. Unblemished polymeric nanofibres or composite nanofibres with unique morphologies and multidimensional congregations going from one-dimensional (1D) to three-dimensional (3D) can be acquired from electrospinning. Basically, these nanofibres have a greater surface-area-to-volume ratio, tuneable porosity and simple surface functionalisation, which present various opportunities for applications, especially in the biomedical field. These are some ongoing advances in electrospinning-based nanomaterials for biomedical applications, for example, antibacterial mats [127], patches for quick haemostasis [128], wound dressings [129] and scaffolds for skin regeneration [130,131].

A few principles should be applied in the scale-up (large-scale) production of nanomaterials for the biomedical field. Among these principles, the quality-by-design (QbD) principle has been used normally in industrial applications such as car manufacturing. For example, it is well known that patient satisfaction is based on the characteristics of nanomaterials, like minimal side effects. At this point, the role of QbD is to remove unwanted side effects from the nanomaterials delivered to the market. There must be a coordinated arrangement between individuals belonging to the industrial sector and scientists; a fruitful group must start with obviously verbalised shared objectives for the item that are quantifiable and approved by healthcare authorities.

### 5.2. Drug and Gene Delivery Systems

Sustained drug delivery in a prolonged manner is desired in numerous situations to achieve enhanced results and enhanced patient consistence; that is why scientists prefer NPs as drug carriers. Numerous research articles have proven this concept. For example, an in vivo study on diabetic rodents demonstrated that subcutaneously infused insulin-stacked polymeric NPs of 85–185 nm size had a longer hypoglycaemic impact than free insulin [105]. In another examination, intramuscularly administered 600 nm PLGA NPs stacked with plasmid DNA demonstrated continued quality delivery [107].

Targeted medication conveyance stands out amongst the most sought-after objectives in medication quality. NPs are fit for focusing on a broad range of stages, from cells to tissues to organs, especially for organelles present in the cells. In general, each tissue and organ has its own transportation mechanism, with a specific size, and most of the time, these transportation mechanisms are different for each tissue and organ. Therefore, targeting a specific tissue or organ can be done by adjusting the particle size, shape and surface functionalisation properties. For instance, veins in the liver contain fenestrations of around 106–175 nm [107]. These organs canthus be specifically targeted by controlling the size and surface properties of the NPs. Numerous kinds of tumours are characterised by permeable veins. The pore cut-off measures these veins somewhere in the range of 380 and 780 nm. Flowing NPs somewhere in the range of 100 and 300 nm would, along these lines, spill through the pores and gather in the tumour due to the enhanced permeability and retention (EPR) effect [107]. In addition, by appending ligands, NPs can effectively target, for all intents and purposes, any sort of available cells with distinguished cell receptors.

To overcome these issues and enhance the remedial impact of cisplatin (CDDP), very specific a supra-portion intra-blood vessel CDDP mixture for cutting-edge Head and neck squamous cell carcinoma (HNSCC) was applied [132]. However, since this method is more confusing than implantation of anti-tumour medications, it is not pervasive in the chemotherapy scene. Of late, a few types of nanoparticle restorative stages, including liposomes, NPs and polymeric micelles, have been created depending on the possibility that the drug delivery system (DDS) can be delivered specifically to the tumour, with decreased circulation in typical tissues and limited undesirable side effects [132,133,134,135]. NC-6004, which is a CDDP-joining polymeric micellar nanoparticle, upgraded anti-tumour movement and diminished the nephrotoxicity and neurotoxicity of CDDP in gastric cancer [136,137,138]. It is a long, well-established reality that a decrease per unit volume of a material builds the surface territory of the material. This essential idea is substantial for medication conveyance advancements using nanoscale elements. When contrasted with regular medication conveyance frameworks, nanotechnology-based medication conveyance frameworks have enhanced surface zones. This expanded surface region per unit volume enhances the stacking and discharging effectiveness of medications.

The nanotechnology-based medication conveyance level provides analysts with choices to deliver exceedingly poisonous medication intermediates and edifices and, in addition, DNA and viral vectors at ideal measurements at controlled interims of time [139]. The release behaviour from materials relies to a great extent on the idea of drug delivery. By picking the right kind of materials for designing NPs, the release profile can be altered [138]. In addition, nanotechnology opens the way to alter the property of materials at the molecular scale, prompting the production of materials with different delivery rates that are, to a great degree, controllable [140]. Nanocarriers may offer a long dissemination time of medications inside the body, while bigger particles will, in general, be expelled a lot quicker than NPs. In other words, bigger particles will, in general, be accumulated in the spleen and liver instead of in target areas. Smaller particles tend to be taken up by different cells inside the body, including target cells [141].

Engineering plays an important role in the delivery of genes using nanomaterials, as conducted using gene guns. Gene guns have been demonstrated to be helpful in conveyance of DNA within the human body [142]. These conveyance devices are basically quickening agents of tiny carrier particles loaded with DNA for direct delivery into tissue-specific places, which ensures perfect transfection of DNA [143]. NPs or submicron particles are commonly required to infiltrate to a specific depth inside the target to complete the ideal impact of quality conveyance, and as such, the infiltration depth of these particles is one of the significant factors examined for the application of gene guns [144].

### 5.3. Challenges, Perspectives and Progress of NPs

The link between the formation and role of NPs is still to be explored. Commonly, NPs adsorb plasma proteins, which subsequently interact with the immune system of the body. Free radicals may be formed because of them, which can likewise cause genotoxicity. The cost of nanomedicines is very high compared to classical treatments due to the use of expensive characterisation instruments. In addition, nanomedicines show an updated stage of safety when contrasted with conventional treatments, yet medical care experts do not ordinarily suggest upgradation in the adequacy level. In general, nanomedicines have an interesting potential to diminish the dose frequency, enhance bioavailability in the light of the decreased size of particles and large surface area. In any case, the fundamental concern is how viable and safe are nanomedicines. Even if any definition makes the pharmacokinetic profile better, nanomedicines, however, produce toxicants inside the body, thus restricting their roles [145,146].

Nanomedicines generally play a significant role in compelling medication delivery. Medication targeting had become more conceivable because of these nanomaterials. Both passive and active targeting should be possible easily [147,148]. Likewise, the expanded surface features bring about proficient medication retention and thus can improve bioavailability. However, the two key difficulties related to them are the immune system response and cytotoxicity, which should be investigated fundamentally. Nanomaterial characterisation is another basic challenge, where various techniques like fluorescence-based tests and cytotoxicity tests are prevented because of the physicochemical properties of nanomaterials. It is suggested that the efficiency of medication loading be emphasised, along with decreasing the delivered doses. Furthermore, it is also recommended that smart nanomaterials that can skip the immune response and do not affect the surrounding environment of the diseased tissue be developed.

## 6. Conclusions

Based on the reviewed literature, it is obvious that nanotechnology has had a significant impact in the biomedical field, prompting extraordinary success in the improvement of an assortment of useful nanomaterials for various medical applications. A few properties of NPs have improved as being the key parameters to control the capacity of the designed NPs, and they ought to be used to progress future endeavours. These properties include the particulate shape, microstructure, diameter and charges in its surface, which can be adjusted by selecting the proper method (e.g., seeding growth technique), materials and preparation tool. Investigation of these properties should be done with a combination of the mentioned characterisation techniques to get a complementary picture about the designed NPs; however, the sample preparation for these investigations is still challenging for some NPs. Therefore, scientists are urged to invent simple and easy characterisation techniques for rapid continuation in this field. Finally, bringing some industrial concepts to the field of biomedical applications based on nanomaterials, such as quality by design (QbD), and encouraging mixed teamwork from industry people and nanomaterial researchers will definitely boost the production scale-up of released products in the market.

## Figures and Tables

**Table 1:** Example references (Refs.) featuring the impacts of typical physico-chemical properties of nanoparticles (NPs) on the uptake of cells.

Physicochemical Properties	Parameter	NPs	Uptake Mechanism	Effects of Important Characteristics	Refs.
**Surface charge**	Positive, negative and neutral	poly(ethylene glycol)-block-polylactic acid PEG-b-PLA	Clathrin- and caveolin-mediated endocytosis	Positive charge improves uptake, transport and distribution	[21]
**Size**	5–100 nm	Ag	Clathrin-mediated endocytosis	Higher uptake of larger NPs; lower cross-plasma membrane becomes faster	[22]
**Shape**	Nanospheres and nanostars	Small interfering RNA-conjugated Au	Endocytosis	Larger spheres (50 nm) and stars (40 nm) show higher uptake	[23]

**Table 2:** Different quantum dot (QD) materials with their emission ranges and sizes.

QD Materials	Emission Range (nm)	QD Size Range (Diameter (nm))	References
Ag_2_S	1000–1300	5.4–10	[41]
PbSe	1110–1310	3.2–4.1	[41,44]
InAs	860–1270	3.2–6	[43]
InP	610–710	2.5–4.5	[41,43]
CdTe/CdSe	650–840	4–9.2	[41,43,44]
CdSe	480–680	2–8	[41,43,44]
CdTe	520–750	3.1–9.1	[41,43,44]
CdS	410–460	2.8–5.4	[41,43,44]

## Data Availability

Not applicable.
